# Tenophages: a novel macrophage-like tendon cell population expressing CX3CL1 and CX3CR1

**DOI:** 10.1242/dmm.041384

**Published:** 2019-12-16

**Authors:** Christine Lehner, Gabriel Spitzer, Renate Gehwolf, Andrea Wagner, Nadja Weissenbacher, Christian Deininger, Katja Emmanuel, Florian Wichlas, Herbert Tempfer, Andreas Traweger

**Affiliations:** 1 Institute of Tendon and Bone Regeneration, Spinal Cord Injury and Tissue Regeneration Center Salzburg, Paracelsus Medical University, 5020 Salzburg, Austria; 2 Austrian Cluster for Tissue Regeneration, 1200 Vienna, Austria; 3 Department of Orthopedics and Traumatology, Paracelsus Medical University, 5020 Salzburg, Austria

**Keywords:** Fractalkine, CX3CL1, CX3CR1, Epiregulin, Macrophage-like, Inflammation, Tendon homeostasis, Tendinopathy

## Abstract

Tendon disorders frequently occur and recent evidence has clearly implicated the presence of immune cells and inflammatory events during early tendinopathy. However, the origin and properties of these cells remain poorly defined. Therefore, the aim of this study was to determine the presence of cells in healthy rodent and human tendon tissue fulfilling macrophage-like functions. Using various transgenic reporter mouse models, we demonstrate the presence of tendon-resident cells in the dense matrix of the tendon core expressing the fractalkine (Fkn) receptor CX3CR1 and its cognate ligand CX3CL1/Fkn. Pro-inflammatory stimulation of 3D tendon-like constructs *in vitro* resulted in a significant increase in the expression of IL-1β, IL-6, Mmp3, Mmp9, CX3CL1 and epiregulin, which has been reported to contribute to inflammation, wound healing and tissue repair. Furthermore, we demonstrate that inhibition of the Fkn receptor blocked tendon cell migration *in vitro*, and show the presence of CX3CL1/CX3CR1/EREG-expressing cells in healthy human tendons. Taken together, we demonstrate the presence of CX3CL1^+^/CX3CR1^+^ ‘tenophages’ within the healthy tendon proper, which potentially fulfill surveillance functions in tendons.

This article has an associated First Person interview with the first author of the paper.

## Abstract (precis)

**Summary:** Here, we demonstrate the presence of a macrophage-like, CX3CL1/CX3CR1-expressing tendon cell population within the healthy tendon proper, which potentially fulfills a surveillance function.

## INTRODUCTION

Tendon pathologies and injuries are one of the most common musculoskeletal disorders; however, due to the tissue's poor regenerative capacity, the healing process is long lasting and outcomes are often not satisfactory. Consequently, tendinopathies represent a substantial social and economic burden (Schneider et al., 2018). The limited availability of effective treatment options not only owes to the multifactorial nature of tendinopathies, but above all results from our insufficient understanding of the cellular and molecular mechanisms leading to the onset and progression of the disease. Therefore, gaining a deeper insight into the nature and function of tendon-resident cells in tissue homeostasis and disease is imperative for developing new treatment strategies for tendinopathies.

Owing to the composition and structure of the extracellular matrix (ECM), tendons are able to withstand enormous tensile forces, so that spontaneous ruptures rarely occur without preceding features of tissue degeneration. Besides repetitive overload, smoking and the intake of certain drugs, obesity and various metabolic diseases are recognized risk factors for the development of tendinopathies. Interestingly, a role of inflammation in the pathogenesis of tendinopathy has long been debated, and the underlying mechanisms are poorly understood. The presence of myeloid and lymphoid cells – such as mast cells, T cells and macrophages – during early human tendinopathy highlight a role of inflammation in tendon disease (Dean et al., 2016; Kragsnaes et al., 2014; Millar et al., 2010). However, the origin of these immune cells is unclear: whether they invade the tissue from the circulation and neighboring tissue, or whether tissue-resident cells are activated upon damage, or a combination of both mechanisms. Generally, tissue-resident macrophages *in vivo* are not a homogeneous cell population, but they are heterogeneous in nature and respond to certain stimuli with overlapping functions and phenotypes; therefore, often cannot be classified into simple, polarized categories (Davies and Taylor, 2015). As the majority of these cells are usually situated in the vicinity of blood vessels (Hume et al., 1984), it seems plausible that this would also apply for tendons. However, the presence and distribution of cells fulfilling macrophage- or monocyte-related functions in healthy tendons has not been thoroughly investigated so far. Owing to the hypovascular nature of tendons, we hypothesize that, in tendons, these cells not only are present in the perivascular region, but also reside within the dense, collagen-rich tendon core, fulfilling a surveillance function similar to that of Langerhans cells in the skin or microglia in the brain (Deckers et al., 2018; Lehner et al., 2016).

In general, the main effectors of inflammation are myeloid cells, most notably monocytes and macrophages. Among the known factors that control e.g. monocyte recruitment is the C-X3-C motif chemokine ligand 1 (CX3CL1), or fractalkine (FKN), and its cognate receptor CX3C chemokine receptor 1 (CX3CR1) (Lee et al., 2018). CX3CR1 is expressed by myeloid and lymphoid lineage cells, including mast cells and natural killer (NK) cells (Mass et al., 2016; Sasmono and Williams, 2012). In addition, CX3CL1/FKN has been demonstrated to regulate the communication between neurons, glia and microglia, and CX3CR1-expressing microglia have been suggested to be pivotal in limiting tissue injury during inflammation and neurodegeneration (Sheridan and Murphy, 2013). Overall, depending on the tissue type, CX3CR1-expressing cells can either contribute to maintenance of tissue homeostasis or play a role in disease progression. These findings prompted us to investigate whether the CX3CL1/CX3CR1 axis might also be relevant in tendons. Therefore, the purpose of this study was to assess the presence of tendon-core-resident cells in healthy rodent and human tissues expressing immune-cell-related markers, and to explore the ramifications of pro-inflammatory stimulation on the CX3CL1/CX3CR1 system in 3D tendon-like constructs *in vitro*.

## RESULTS

### Tendon-resident cells express immune-cell-related markers

**Fig. 1.**
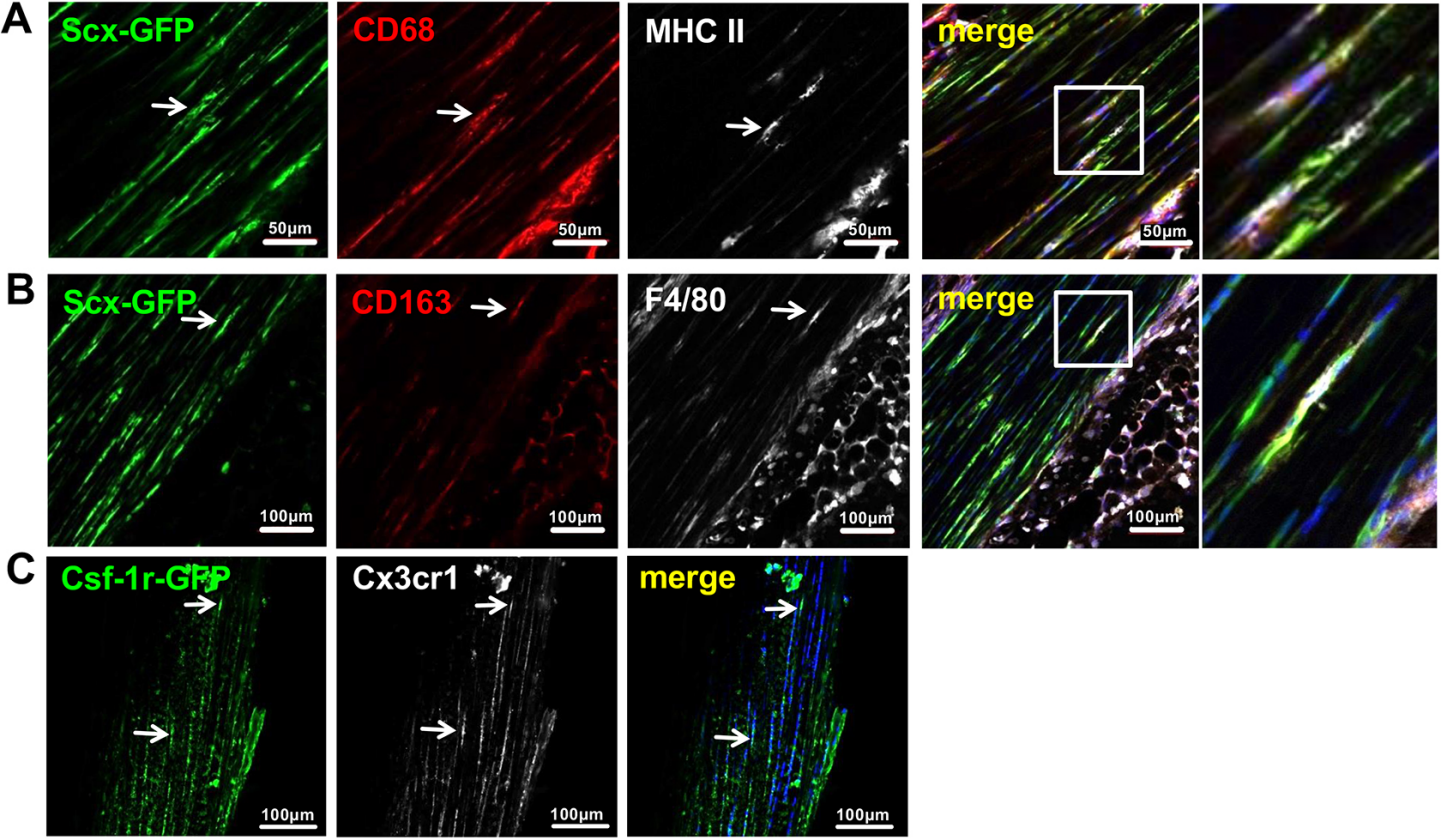
**Expression of immune cell markers in mouse tendon.** (A-C) Immunohistochemical staining of immune cell markers on histological sections of Achilles tendons from *Scx-GFP* transgenic mice reveals that Scx-positive cells co-express CD68, MHCII, CD163 and F4/80 (A,B; arrows). Cryosections of Achilles tendon from transgenic *Csf-1r* reporter mice show that cells within the dense part of the tendon are positive for CSF-1R and CX3CR1 (C; arrows).

**Fig. 2.**
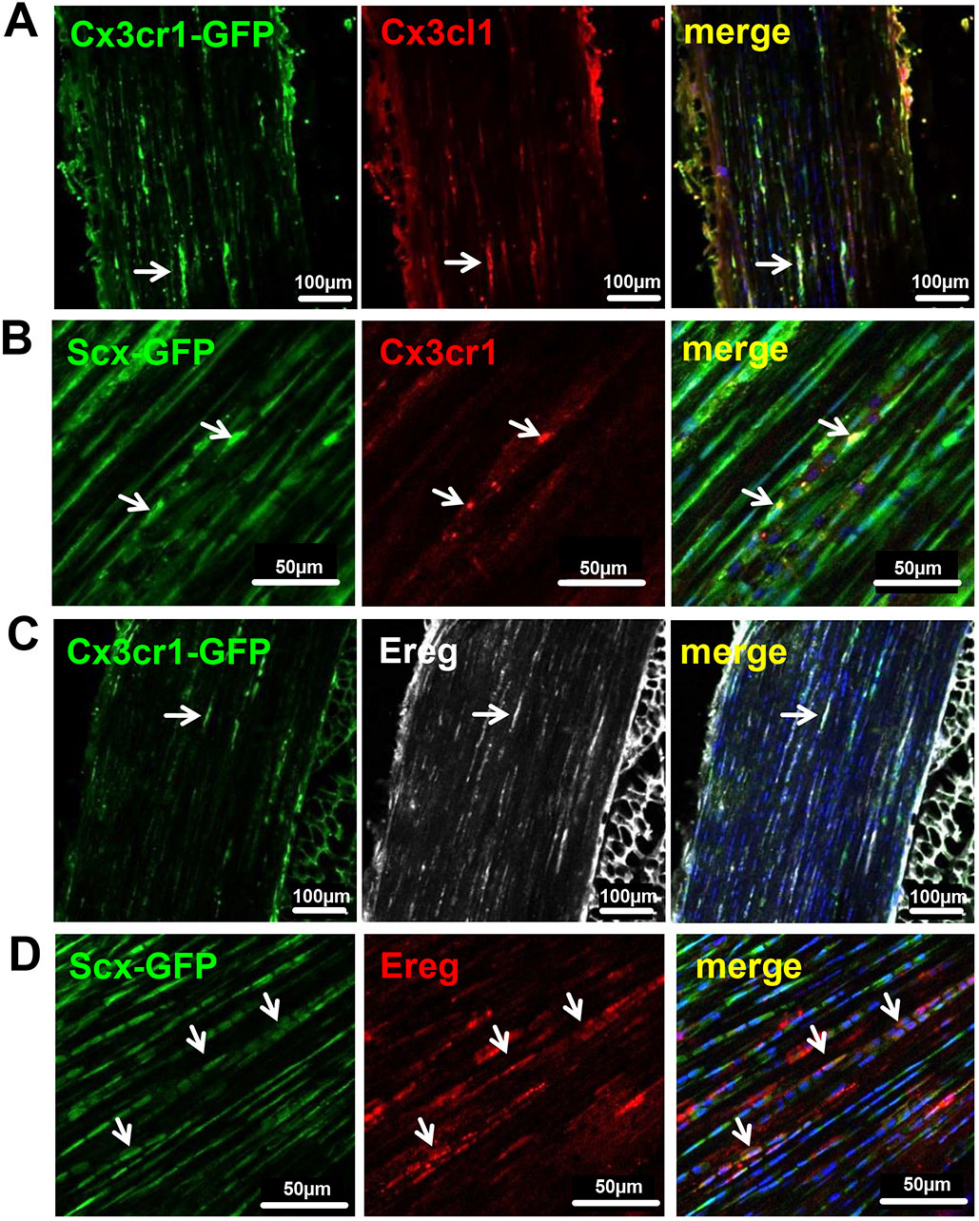
**Expression of CX3CL1, CX3CR1 and Ereg in tendons of *Scx-GFP* and *Cx3cr1-GFP* mice.** (A-D) Cryosections of Achilles tendons from transgenic *Cx3cr1-GFP* (A,C) and *Scx-GFP* (B,D) reporter mice immunohistochemically stained with antibodies recognizing CX3CL1/FKN, its receptor CX3CR1 and Ereg. Arrows point towards cells co-expressing the respective proteins.

To evaluate the presence of tendon-resident cells expressing immune cell markers, we probed Achilles tendon tissue sections from the transgenic *Scx-GFP* tendon reporter mouse strain (Pryce et al., 2007). As shown in Fig. 1A and B, GFP-positive cells located in the dense tendon core co-expressed the widely used pan-macrophage marker CD68, F4/80 (also known as Adgre1), a unique marker of murine macrophages, and the macrophage-specific hemoglobin scavenger receptor cluster of differentiation 163 (CD163). Further, immunohistochemical staining revealed tendon cells co-expressing major histocompatibility complex II (MHCII; also known as HLA-DRB1), a membrane-bound marker for antigen-presenting cells, such as macrophages, B lymphocytes and dendritic cells (Kristiansen et al., 2001). To further substantiate the presence of macrophage-like cells in the tendon proper we also investigated Achilles tendon tissue of the transgenic MacGreen reporter mouse strain. These mice express EGFP under the control of the mouse colony stimulating factor 1 receptor (*Csf-1r*) promoter, labeling mononuclear phagocyte lineage cells (Sasmono et al., 2003). As shown in Fig. 1C, several cells in the tendon proper were positive for EGFP, indicating the presence of potentially phagocytic cells. Further, the majority of the EGFP-positive cells also stained positive for CX3CR1, and expression of the receptor was confirmed using a transgenic mouse strain expressing EGFP driven by the *Cx3cr1* promoter (Jung et al., 2000) (Fig. S1). Finally, by employing double immunolabeling, we further demonstrate that CX3CR1 and its ligand CX3CL1 are both co-expressed by tendon cells (Fig. 2A). The expression of CX3CR1 specifically in tendon cells was also confirmed by probing Achilles tendon sections of the *Scx-GFP* tendon reporter mouse strain (Fig. 2B).

FKN has been described to induce shedding of epiregulin (EREG), a 46-amino-acid protein belonging to the epidermal growth factor (EGF) family of peptide hormones, and further to rapidly increase *EREG* mRNA expression 20-fold (White et al., 2010). Therefore, we investigated tendon tissue sections for the presence of Ereg. Indeed, Ereg is also expressed in tendon-resident cells expressing Scx-GFP or CX3CR1-EGFP (Fig. 2C,D). Finally, CX3CR1-positive cells also express macrophage markers CD68 and CD163 (Fig. S2).

**Fig. 3.**
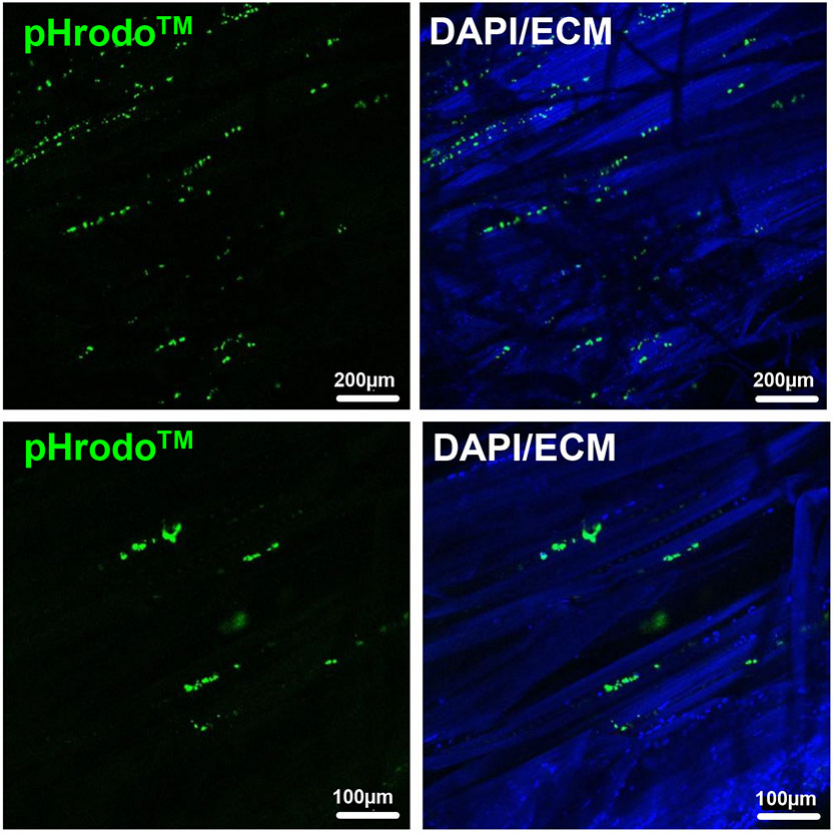
***In situ* tendon cell phagocytosis assay.** *In situ* phagocytosis assay on unfixed rat flexor tendons shows that tendon cells lying within the dense collagen matrix [shown by extracellular matrix (ECM) autofluorescence/blue channel] exert phagocytic activity (green fluorescence). Two representative regions are shown.

Next, to determine whether these cells, apart from their macrophage-associated marker profile, also possess phagocytic activity, we exposed unfixed rat flexor tendons to pHrodo™ Green *S. aureus* Bioparticles™, which upon cellular uptake emit fluorescence due to a shift in pH. As shown in Fig. 3, we detected several positive cells within the tendon core embedded in the dense collagenous matrix, demonstrating the presence of phagocytic cells within the tendon proper *in vivo*.

### Pro-inflammatory stimulation of 3D tendon-like constructs increases Fkn and Ereg expression

**Fig. 4.**
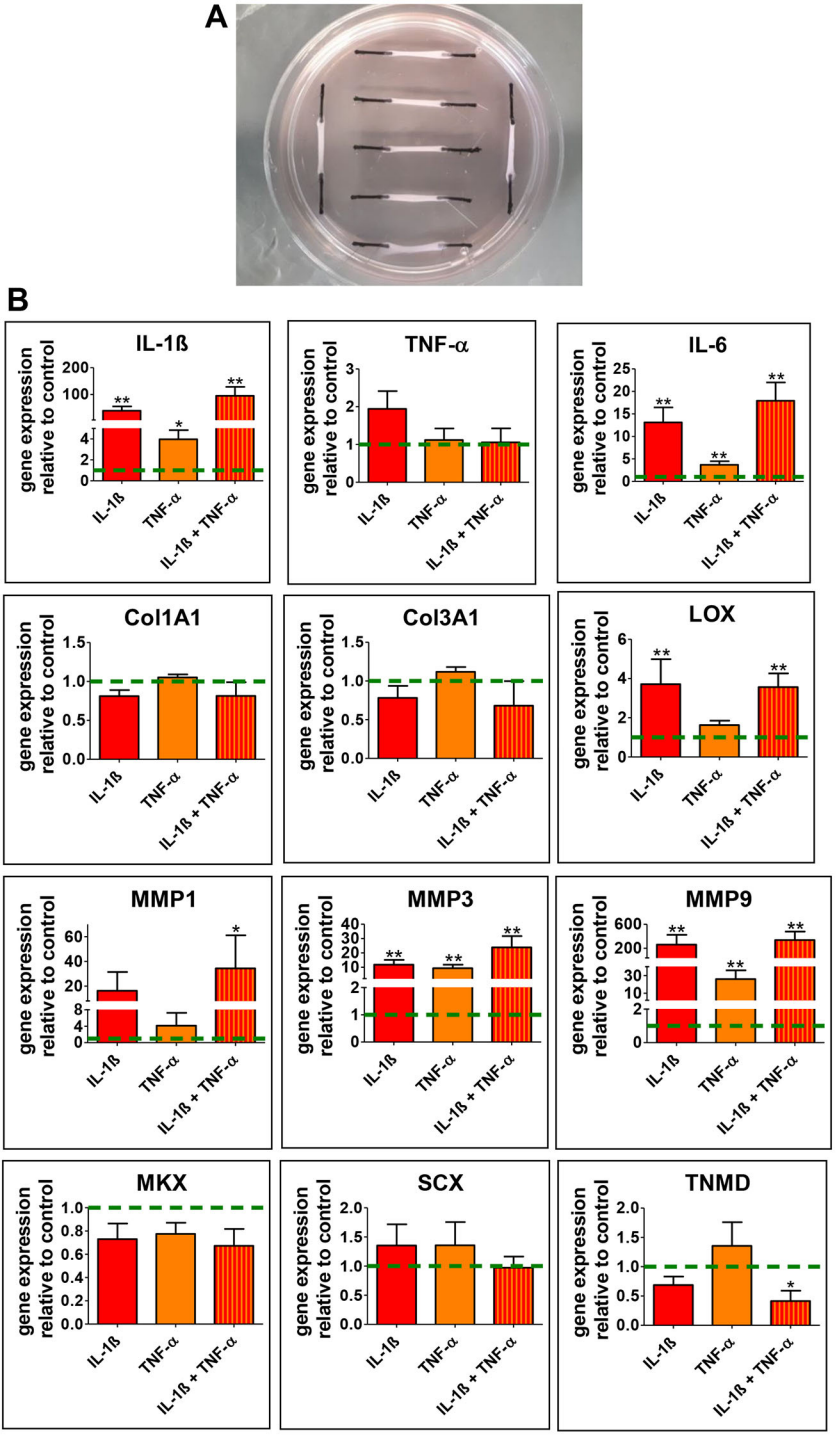
**qRT-PCR analysis of tendon-like constructs.** (A) 3D tendon-like constructs were stimulated with either IL-1β, TNF-α or a combination of both cytokines. (B) Effects on the expression levels of genes encoding inflammatory proteins (*Il-1b*, *Tnf-α*, *Il-6*), ECM-related proteins (e.g. *Col1a1*, *Col3a1*, *Lox*, *Mmp1*, *Mmp3*, *Mmp9*) and those encoding tendon-cell-related marker proteins (*Mwk*, *Scx*, *Tnmd*) were assessed by qRT-PCR. Significant changes were detected for *Il-1b*, *Il-6*, *Lox*, *Mmp1*, *Mmp3* and *Mmp9.* Bars represent mean±s.e.m. (for five individual animals); *P<0.05, **P<0.01, Mann–Whitney test. Dashed green line, control reference.

Having identified tendon-resident cells expressing immune cell-related markers, we next examined the response of primary tendon stem and progenitor cells (subsequently referred to as TDSPCs) to pro-inflammatory stimuli. We therefore generated 3D type I collagen-embedded tendon cell cultures as previously described (Gehwolf et al., 2019), and analyzed the expression of tendon-specific and matrix-associated as well as inflammation-related markers after exposure to IL-1β, TNF-α or a combination of both (Fig. 4A). As shown in Fig. 4B, stimulation of the constructs significantly increased the gene expression of *Il-1b*, *Tnf-a* and *Il-6*, as well as the expression of genes encoding extracellular matrix (ECM)-associated proteins such as lysyloxidase (*Lox*) and the matrix metalloproteinases (MMPs) *Mmp1*, *Mmp3* and *Mmp9*. A synergistic effect of Il-1β and TNFα stimulation was seen for several candidate genes; however, IL-1β treatment generally had a more pronounced effect on gene expression. No significant effect was evident for the expression of genes encoding type I collagen (*Col1a1*) and type 3 collagen (*Col3a1*). Further, there was little or no impact on the expression of the genes encoding tenogenic marker proteins tenomodulin (*Tnmd*), mohawk (*Mkx*) and scleraxis (*Scx*).

**Fig. 5.**
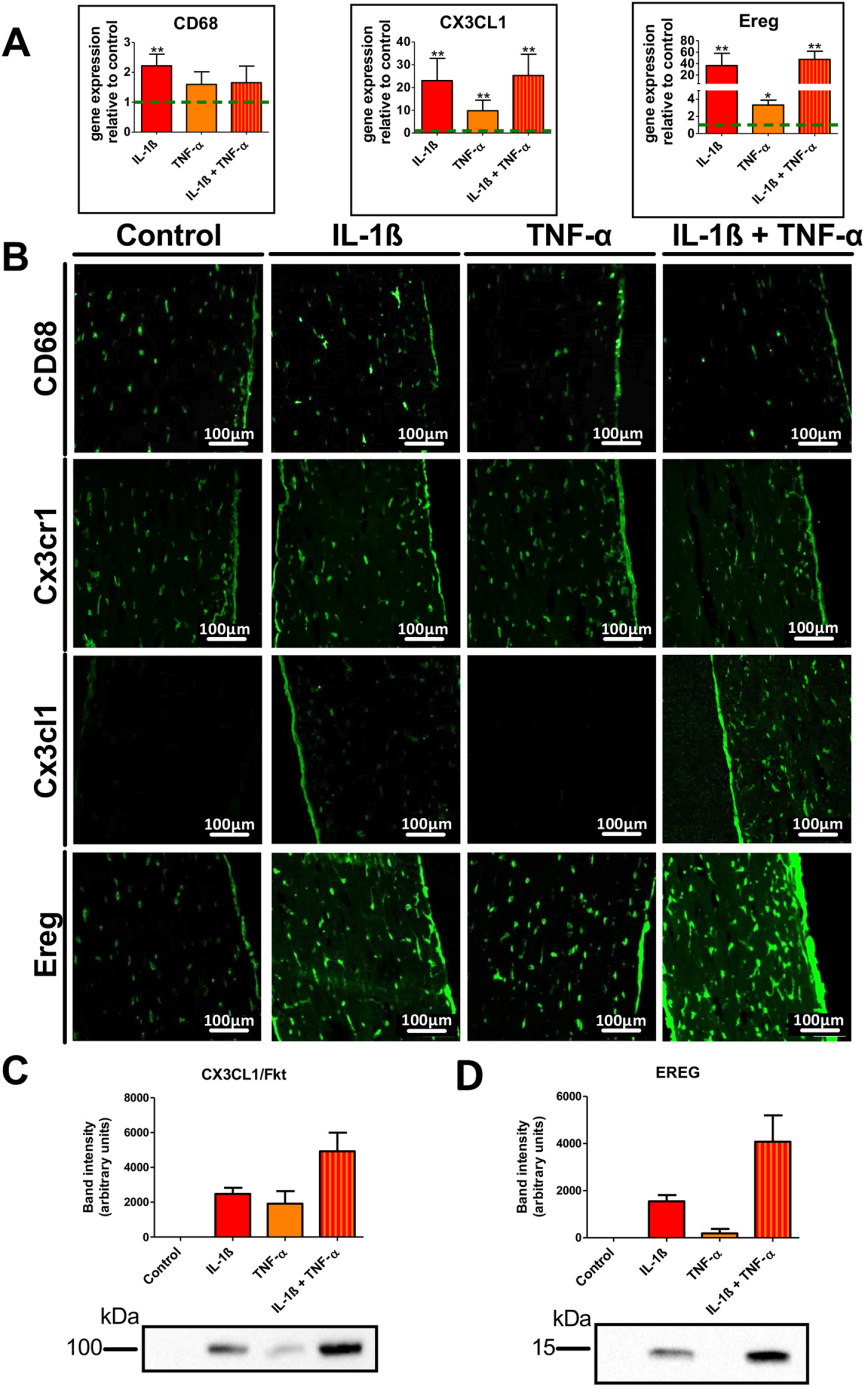
**Pro-inflammatory stimulation of tendon-like constructs.** (A-D) Effects of pro-inflammatory stimulation of tendon-like constructs on mRNA (A) and protein (B,C). IL-1β or TNF-α or a combination of both cytokines resulted in a significant upregulation of *Cd68*, *Cx3cl1* and *Ereg* mRNA expression (A). Immunohistochemical staining confirmed the qRT-PCR findings. CX3CR1 remained unaffected by the treatment (B). Furthermore, western blot analysis revealed a synergistic effect of IL-1β and TNF-α on CX3CL1 (C) and Ereg (D) expression. *P<0.05, **P<0.001, Mann–Whitney test.

IL-1β exposure led to a moderate 2-fold increase in the expression of the macrophage-related marker CD68, whereas a significant increase (≥20-fold) in *Fkn* (*Cx3cl1*) and *Ereg* mRNA quantities was observed, which was even higher if co-stimulated with TNF-α. These results were further underscored by immunofluorescent analysis, demonstrating that pro-inflammatory treatment mainly affected the expression of CX3CL1 and Ereg (Fig. 5B). Finally, to obtain quantitative data on protein levels, we also performed western blot analysis on lysates prepared from stimulated and unstimulated 3D tendon-like constructs. Again, a significant increase in expression was observed for both CX3CL1 and Ereg (Fig. 5C).

### Inhibition of CX3CR1 signaling blocks tendon cell migration

**Fig. 6.**
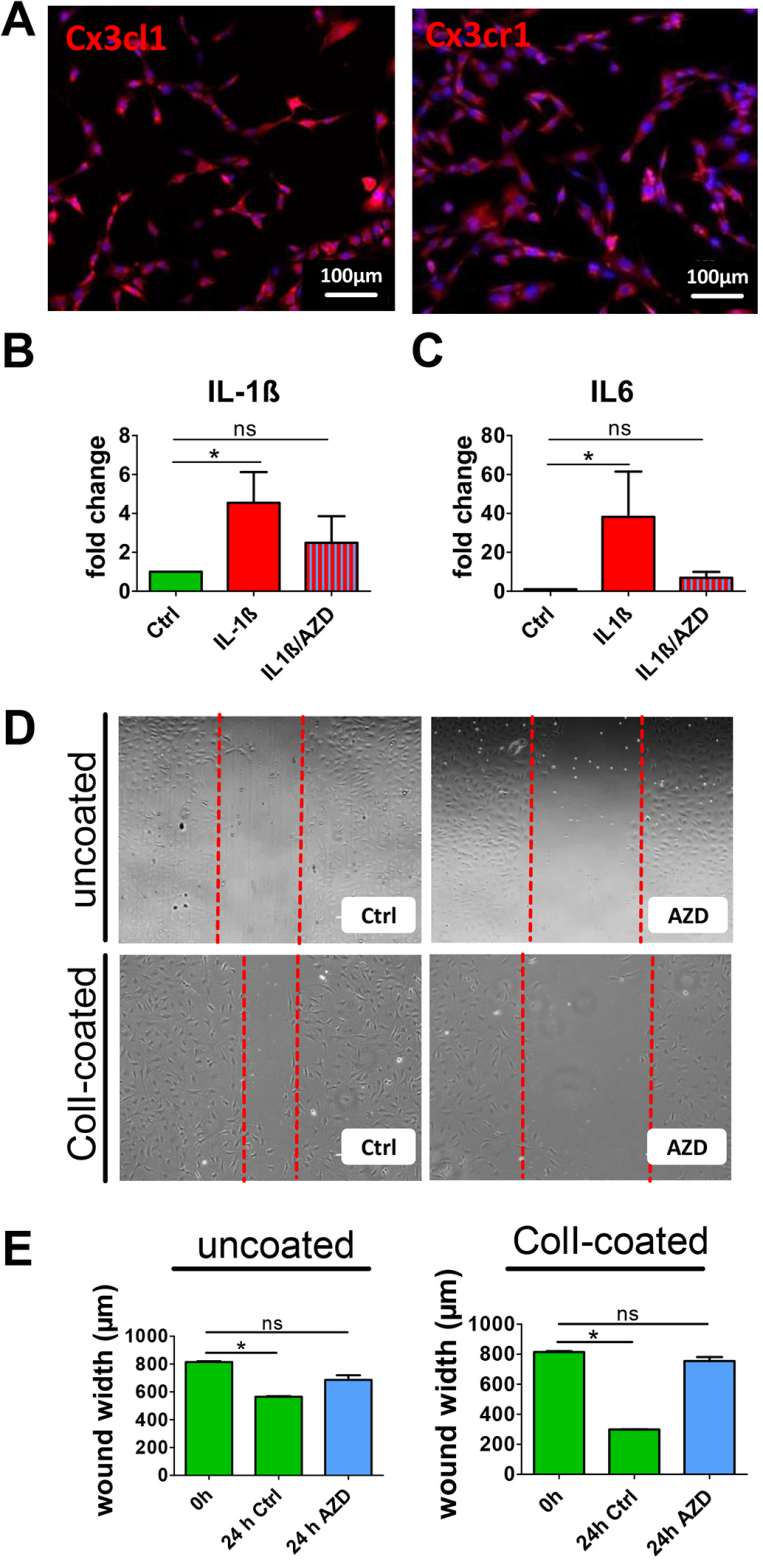
**Effects of CX3CR1 inhibition on tendon cells.** (A) Rat tendon-derived cells (passage 1) express CX3CL1 and CX3CR1. (B,C) Addition of AZD 8797 attenuates IL-1β-triggered upregulation of both IL-1β (B) and IL-6 (C). (D) Representative images showing wound scratch assays on uncoated and collagen-coated culture plates; the dashed red lines mark the front of the migrating cells. (E) Quantitative analysis revealed that the FKN inhibitor AZD 8797 significantly reduces migration. ns, non-significant; *P<0.05, Kruskal–Wallis and Dunn's multiple comparison test. Ctrl, control.

In order to address a putative function of the CX3CL1/CXCR1 signaling axis in tendon-resident cells, we next performed cell migration assays. To inhibit CX3CR1, we applied AZD 8797 (Axon Medchem, Groningen, The Netherlands), a selective, high-affinity small-molecule inhibitor of CX3CR1. Importantly, early-passage rat TDSPCs (p1) retain the expression of both Fkn and its receptor (Fig. 6A). Interestingly, treatment with the Fkn receptor antagonist led to a reduction of IL-1β-triggered mRNA expression of *Il-1b* and *Il-6* to control levels (Fig. 6B**,**C). Analysis of the wound scratch assay revealed that AZD 8797 almost completely blocked migration of TDSPCs on uncoated and type I collagen-coated cell culture dishes, whereby the effect was stronger on the collagen-coated dishes (Fig. 6D,E).

### CX3CL1, CX3CR1 and EREG are expressed in healthy human tendon tissue

**Fig. 7.**
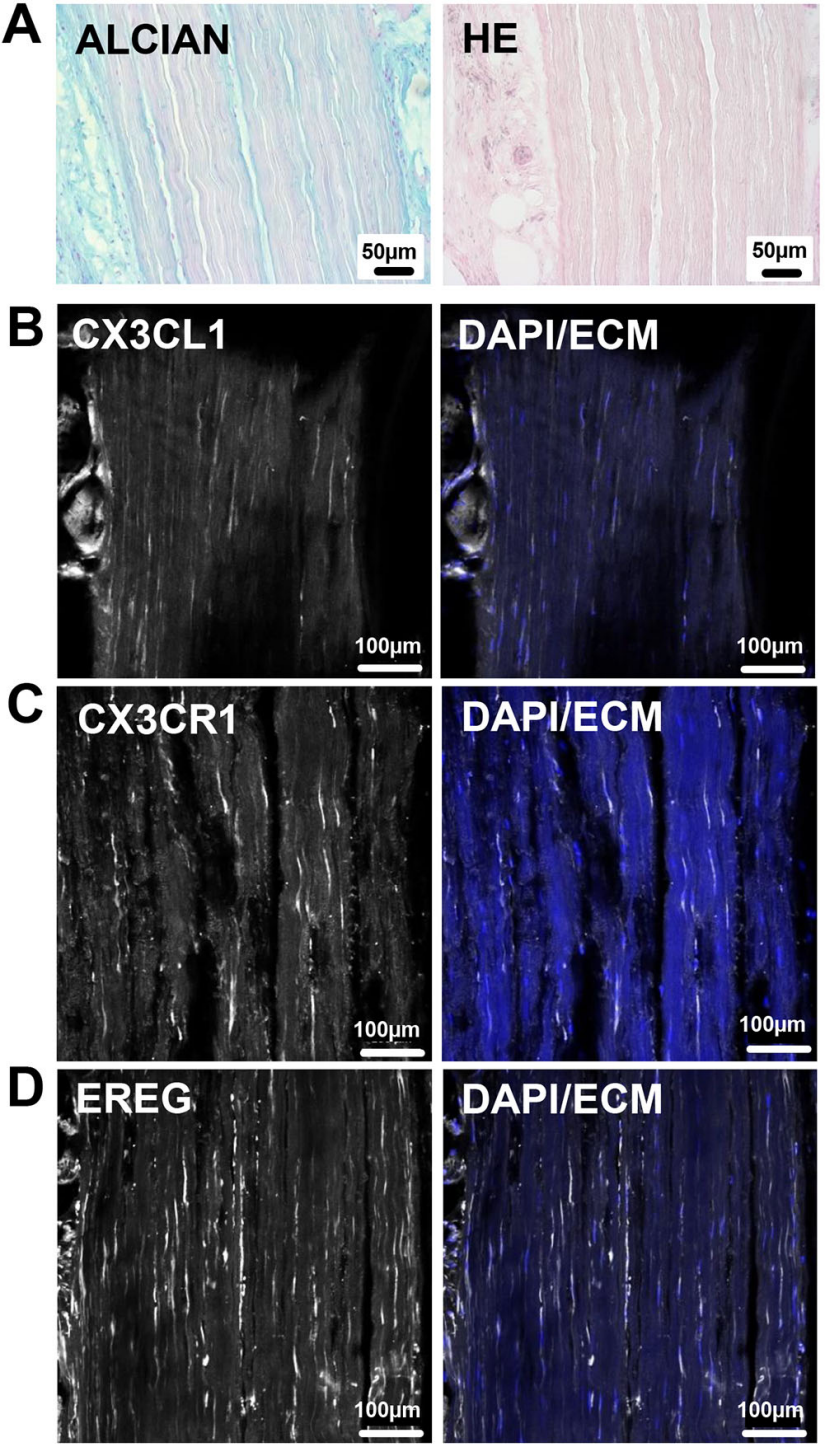
**Expression of CX3CL1, CX3CR1 and EREG in intact human tendon.** Cryosections of intact human semitendinosus tendon (male, aged 34 years). (A) Alcian Blue and Hematoxylin and Eosin (HE) staining shows the parallel alignment of collagen fibers and elongated cell nuclei characteristic of intact tendons. (B-D) Immunofluorescent images demonstrating the presence of cells expressing CX3CL1/FKN (B), its cognate receptor CX3CR1 (C) and EREG (D) in the tendon proper.

Finally, we were interested to see whether FKN, its receptor CX3CR1 and EREG are also expressed in healthy human tendons. To this end, we probed cryosections of human semitendinosus tendons obtained from a healthy, 34-year-old male (Fig. 7A). Indeed, next to strong expression at blood vessel walls (Fig. S3), our analysis revealed the presence of distinct cells within the tendon proper, expressing CX3CL1, CX3CR1 and EREG (Fig. 7B-D). To conclude, our results clearly demonstrate the presence of a CX3CL1/CX3CR1/EREG-expressing cell population in healthy murine and human tendon tissue.

## DISCUSSION

Our understanding of the cellular and molecular mechanisms underlying tendinopathies remains very fragmentary. The term tendinopathy encompasses a broad spectrum of tendon-related diseases and is mainly characterized by activity-related pain. Historically, there has been substantial debate about the terminology and whether inflammation is of importance in the development and progression of tendinopathies (Khan et al., 2002, 2000). In contrast, more recent studies elegantly highlight the involvement of immune cells and activation of inflammatory processes in tendinopathy (Dean et al., 2017). However, the origin of these cells remains unknown, and it is unclear if they mainly extravasate into the tissue upon injury or metabolic stress, or if tendon-resident cells exist in healthy tendon tissue fulfilling macrophage- or mast cell-like functions. Therefore, we aimed to formally demonstrate the presence of ‘tenophages’ in the tendon core of intact, healthy murine and human tendons.

In the present study, we demonstrate the presence of cells positive for immune-cell-related markers located within the dense tendon core region (Fig. 1). Interestingly, these cells were also positive for the widely accepted tendon-specific marker Scx, a member of the basic helix-loop-helix (bHLH) superfamily of transcription factors. Hence, it is indeed tendon cells themselves expressing immunocyte-related surface marker proteins. To our knowledge, this is the first description of such a cell population in healthy tendons. Apart from their cell surface marker profile, these cells also exert phagocytic activity as evidenced by an *ex vivo* phagocytosis assay (Fig. 3). Remarkably, other musculoskeletal cell types including articular chondrocytes, chondrogenic progenitors or osteoblasts have been described to display macrophage-like behavior, such as enhanced phagocytosis *in vitro* (Kurdziel et al., 2017; Lohmann et al., 2000; Zhou et al., 2016). The presence of such a macrophage-like cell population capable of phagocytosis may serve as a first-line response to small-scale damage by immediately removing cellular debris and initiating an inflammatory response. Further, by making use of a transgenic mouse model we demonstrate the presence of a population of cells within the tendon proper expressing Fkn (CX3CL1) and its cognate receptor CX3CR1 (Fig. 2). CX3CR1 expression is associated with increased cell migration and site-specific dissemination and has been shown in endothelial cells, mast cells, monocytes, tissue-resident macrophages, NK cells, microglial cells, neurons and subpopulations of T lymphocytes (Imai et al., 1997; Papadopoulos et al., 2000; You et al., 2007).

The seven-transmembrane-domain G-protein-coupled CX3CL1 receptor CX3CR1 mediates several intracellular signaling pathways, such as p38MAPK (also known as MAPK14) signaling and the Akt pathway (Li et al., 2016; Wu et al., 2016). It has two known functional ligands, the chemokine CX3CL1 (also called neurotactin or FKN) and CCL26 (eotaxin-3), the latter being 10-fold less potent than CX3CL1 (Nakayama et al., 2010). FKN is structurally unique amongst the family of chemokines and is expressed in the central nervous system and peripheral nerves, as well as in endothelial cells, dendritic cells and lymphocytes (Bazan et al., 1997; Kanazawa et al., 1999; You et al., 2007). It is constitutively cleaved by the ADAM metalloprotease ADAM10, and, upon cell stress, such as tissue injury, shedding is further promoted by ADAM17 (also known as TNF-α-converting enzyme, TACE), releasing an extracellular soluble fragment. In its soluble form, FKN mediates chemotaxis of immune cells, whilst membrane-bound FKN acts as an adhesion molecule mediating leukocyte capture and infiltration (Clark et al., 2011; Imai et al., 1997; Umehara et al., 2004). FKN has been reported to be released by apoptotic lymphocytes stimulating macrophage chemotaxis and recruiting professional phagocytes to the site of cell death (Truman et al., 2008). Beyond simple recruitment, FKN has also been shown to enhance the ability of macrophages and microglia to execute their phagocytic functions (Tsai et al., 2014). Since accumulation of microruptures preceding tendon tears goes along with cell death and subsequent clearance of the cellular debris is required, it is tempting to speculate that the presence of FKN in the tendon might serve as a ‘find-me’ signal for macrophages invading the tissue from the circulation (Lundgreen et al., 2011; Sokolowski et al., 2014).

The CX3CL1/CX3CR1 axis plays a pivotal role in the central nervous system (Liu et al., 2017; O'Sullivan and Dev, 2017; Wu et al., 2016). In an extensive review, Sheridan and Murphy (2013) highlight the crosstalk of neurons and glia in health and disease and discuss that the FKN/CX3CR1 ligand/receptor pair seems to have evolved as a communication link between neurons and microglial cells, being crucial not only for maintaining tissue homeostasis under normal physiological conditions, but also being activated under inflammatory conditions such as stroke or Alzheimer's disease. We speculate that the observed presence of the CX3CL1/CX3CR1 system within the tendon might serve similar surveillance functions as in the brain and that, upon inflammatory stimulation, the system reacts by upregulating FKN, thereby attracting additional monocytes from the circulation.

Besides inducing migration of osteoarthritis fibroblasts, FKN has been shown to act as an angiogenic mediator *in vitro* and *in vivo* (Klosowska et al., 2009). FKN not only significantly induced migration both of human umbilical vein endothelial cells as well as bovine retinal capillary endothelial cells, but also promoted formation of endothelial cell capillary tubes on synthetic matrix and blood vessel growth in a rabbit corneal pocket neovascularization assay (You et al., 2007). These observations of the pro-migratory effect of FKN corroborate our own data, showing that addition of the CX3CR1-specific inhibitor AZD 8797 results in significantly reduced migration of rat tendon-derived cells *in vitro* (Fig. 6).

Next to promoting cell migration, FKN also enhances proliferation. In osteoarthritis fibroblasts, FKN has been shown to induce aortic smooth muscle cell proliferation through an autocrine pathway (Klosowska et al., 2009; White et al., 2010). Interestingly, the observed effects of FKN on proliferation of coronary artery smooth muscle cells (CASMCs) are accompanied by transcription and release of EREG. In their study, White et al. describe that FKN induces shedding of EREG and increases *EREG* mRNA expression 20-fold within 2 h (White et al., 2010). Here, we report the presence of Scx-positive tendon cells also expressing CX3CR1 and Ereg. EREG is a 46-amino-acid protein belonging to the EGF family of peptide hormones. It binds to EGF receptors (EGFR) ErbB1 (HER1) and ErbB4 (HER4) and can stimulate signaling of ErbB2 (HER2/Neu) and ErbB3 (HER3) through ligand-induced heterodimerization with a cognate receptor. EREG is initially expressed as an extracellular transmembrane protein, which is cleaved by disintegrins and metalloproteinase enzymes (ADAMs), releasing a soluble form. It has been shown to contribute to inflammation, wound healing, tissue repair and oocyte maturation by regulating angiogenesis and vascular remodeling, and by stimulating cell proliferation as well as cell migration (Cao et al., 2018; Harada et al., 2015; Martin et al., 2017; Murakami et al., 2013; Riese and Cullum, 2014; Zhuang et al., 2007). Further, in Caco-2 epithelial cells *EREG* mRNA and protein levels have been shown to be increased by incubation with exogenous IL-1β (Massip-Copiz et al., 2018). This finding is well in line with our own data revealing that stimulation of 3D tendon-like constructs with IL-1β, or a combination of IL-1β and TNF-α significantly increased the expression of EREG, both at gene as well as protein level.

Recently, it has been shown that nuclear factor kappaB (NF-κB) signaling is increased in clinical tendinopathy, which is particularly interesting against the background that FKN is stimulated by NF-κB-mediated inflammatory processes (Abraham et al., 2019). Garcia et al. (2000), for example, showed that NF-κB-dependent FKN induction in rat aortic endothelial cells is stimulated by IL-1β, TNF-α and lipopolysaccharide. Moreover, in human lung fibroblasts, a dramatic increase in both soluble CX3CL1 protein and mRNA transcripts in a dose- and time-dependent manner has been reported to be synergistically induced by a combination of IL-1β and IFN-γ (Isozaki et al., 2011). Again, we observed similar responses in 3D tendon cell cultures upon stimulation with IL-1β, TNF-α or a combination of both (Fig. 5).

In addition to its role in angiogenic, migratory and proliferative processes, the CX3CL1/CX3CR1 axis has also been demonstrated to play a role in fibrosis and wound healing in skin, liver and kidney (Arai et al., 2013; Clover et al., 2011; Ishida et al., 2008; Song et al., 2013; Wasmuth et al., 2008). These outcomes are not only due to increased CX3CR1-mediated recruitment of monocytes, but are also a consequence of increased cell proliferation and ECM production of local tissue macrophages. However, reports on the role of CX3CR1 in tissue fibrosis are contradicting. Whereas Engel et al. (2015) report that CX3CR1 deficiency enhances renal fibrosis, Song et al. (2013), in their study on the role of the CX3CL1/CX3CR1 system in diabetic nephropathy, show that markers of renal inflammation, fibrosis and ECM (such as the fractional mesangial area, fibronectin and collagen) were significantly lower in diabetic CX3CR1 knockout (KO) mice compared to diabetic wild-type (WT) mice. Studies in the skin attribute an important role to the CX3CL1/CX3CR1 system in healing processes, demonstrating that CX3CR1 deficiency resulted in delayed wound closure due to reduced myeloid cell recruitment, a marked reduction of macrophage-released products, such as TGF-β1 and vascular endothelial growth factor. Further, reduced alpha-smooth muscle actin (a marker of myofibroblasts), collagen deposition and subdermal angiogenesis have been observed (Clover et al., 2011; Ishida et al., 2008). Based on these and our own findings, we speculate that the presence of CX3CR1-expressing tenophages might also play a role in tendon healing by modulating proliferative, angiogenic and fibrotic responses upon tissue injury.

In summary, we describe the presence of macrophage-like tendon cells (‘tenophages’) and provide evidence for the expression of the CX3CL1/CX3CR1 axis and the peptide hormone epiregulin in healthy rodent as well as human tendons. Interestingly, not only did we observe perivascular expression of these proteins, but also very distinctly in cells within the dense, collagen-rich matrix of tendons. We therefore propose that this newly identified cell population fulfils a surveillance function and is activated upon tendon tissue injury or pathological stress. Given the role in cell proliferation, angiogenesis and fibrosis upon inflammation, and considering that they are hallmarks of tendinopathy, targeting the CX3CL1/CX3CR1/EREG axis could potentially open up new vistas in tendinopathy therapy.

## MATERIALS AND METHODS

### Cell culture

Primary TDSPCs were isolated from the Achilles tendons of five rats (Fisher, female, aged 12 weeks). To this end, rat Achilles tendons were dissected, finely minced and digested in Dulbecco's modified Eagle's medium (DMEM) containing 2 mg/ml type II collagenase (Sigma-Aldrich, St. Louis, MO, USA) for 12 h at 37°C and 5% CO_2_. The isolated cells were placed in DMEM containing 10% fetal bovine serum (FBS), 100 units/ml penicillin and 100 μg/ml streptomycin, at 37°C with 5% CO_2_. Only passages 1-3 of the obtained TDSPCs were used in this study. Results of at least three independent experiments are presented.

### Tendon-like constructs

In order to better mimic the tendon's natural environment, we performed most of our experiments using 3D-collagen-embedded tendon cell cultures. These artificial tendon-like constructs were established as described by our group (Gehwolf et al., 2019). In brief, 2.5×10^5^ rat Achilles tendon-derived cells (passage 2) were mixed with collagen type I (PureCol™ EZ Gel solution, 5074, Sigma-Aldrich, Vienna, Austria; end concentration 2 mg/ml) and spread between two silk sutures pinned with insect pins in rows on SYLGARD 184 (Sigma-Aldrich)-coated Petri dishes. To improve formation of the constructs, aprotinin, ascorbic acid and L-proline were added to the cell culture medium. After contraction of constructs over the course of 11 days, 10 ng/ml IL-1β (PeproTech, Vienna, Austria), 10 ng/ml TNF-α (Invitrogen, Carlsbad, CA, USA) and a combination of both cytokines, respectively, was added to the culture medium. After incubation for 24 h, constructs were harvested and stored either in TRIReagent (Sigma-Aldrich) for further quantitative reverse transcription PCR (qRT-PCR) analysis, fixed in 4% paraformaldehyde for immunohistochemical analysis or frozen at −80°C for subsequent western blot analysis.

### Animals

All procedures involving animals were carried out in an approved animal facility by authorized staff and were in accordance with Austrian laws. As only tissue from euthanized animals was used, no further ethics approval was required. C57BL/6 mice (males, aged 10-12 weeks, 20-25 g) were purchased from the Charles River Laboratories (Wilmington, MA, USA). All animals were acclimatized to standard laboratory conditions (14-h light, 10-h dark cycle) and given free access to rodent chow and water.

*Cx3cr1*-*GFP* and *Csf-1r*-*GFP* transgenic mice were kindly provided by Dr Stella Autenrieth from the Medical Clinic of the University of Tübingen, Tübingen, Germany, and Prof. Thomas Langmann from the Eye Clinic of the University of Cologne, Cologne, Germany, respectively.

Female, 12-week-old Fisher rats were purchased from Janvier Labs (Le Genest-Saint-Isle, France).

### Human tendon tissue

Human semitendinosus tendons available in the course of cruciate ligament reconstructions were provided by the local university clinic after ethics approval (E-Nr. 2374) by the local government and prior patients' informed consent.

### Preparation of tissue sections

Mouse Achilles, human semitendinosus tendons and rat tendon-like constructs were fixed in 4% paraformaldehyde for 12 h at 4°C, and, after several washes in phosphate-buffered saline (PBS) and cryopreservation in 30% sucrose in PBS, embedded in cryomedium (Surgipath Cryogel^®^, Leica Microsystems, Vienna, Austria). Subsequently, 12 µm cryosections were prepared using a Leica CM1950 cryostat.

### Histology and immunohistochemistry

For descriptive histology, cryosections were stained using Hematoxylin and Eosin or Alcian Blue, according to standard protocols. In brief, after staining the sections with Weigert Hematoxylin for 2 min, the staining was stopped with 1% acetic acid, including a short differentiation step by shortly dipping the slides into HCl/ethanol. After blueing the sections under running tap water for 10 min, sections were stained with 1% Eosin Y solution for 1 min and again immersed in 1% acetic acid to stop the staining reaction. Subsequently, the sections were dehydrated in an increasing ethanol series (70%, 96%, 2×100%) and incubated twice in Roti-Histol (Carl Roth, Karlsruhe, Germany). Finally, sections were coverslipped with mounting medium.

For Alcian Blue staining, sections were incubated in Alcian Blue solution (pH 2.5) for 15 min, rinsed in tap water and counterstained with Neutral Red for 1 min. Finally, sections were rapidly dehydrated in absolute alcohol, cleared in Roti-Histol and mounted in Roti-Histokitt (Carl Roth).

In order to demonstrate that tendon cells do express macrophage-like markers we used various antibodies, so as not to rely on a single marker. For identification of immune-cell-related markers within the tendon, we selected well-accepted macrophage markers described to be present in a broad variety of tissue macrophages and also in perivascular macrophages such as CD68 (the mouse equivalent termed macrosialin) and F4/80. The transmembrane glycoprotein CD68 has been shown to be particularly useful for the various cells of the macrophage lineage, including monocytes, histiocytes, giant cells, Kupffer cells and osteoclasts. Moreover, we applied the high-affinity scavenger receptor for the hemoglobin–haptoglobin complex CD163, CX3CR1 and MHCII, further widely used markers of cells from the monocyte/macrophage lineage.

Immunohistochemical detection of immune-cell-related markers was performed on cryosections of tendons and tendon-like constructs. After a 5-min rinse in Tris-buffered saline (TBS; CarlRoth) slides were incubated for 1 h at room temperature (RT) in TBS containing 10% donkey serum (Sigma-Aldrich), 1% bovine serum albumin (BSA; Sigma-Aldrich) and 0.5% Triton X-100 (Merck, Darmstadt, Germany). After a 5-min rinse, slides were subsequently incubated for double or triple immunohistochemistry (overnight at 4°C) with antibodies directed against FKN/CX3CL1 (ab25088, Abcam, Cambridge, UK; 1:100), CX3CR1 (orb10490, Biorybt, Cambridge, UK; 1:100), CD68 (sc20060, Santa Cruz Biotechnology, Dallas, TX, USA; 1:50), CD163 (ab182422, Abcam; 1:100), EREG/aa1-162 (LS-C314859, LSBio, Seattle, WA, USA; 1:100; ab195620, Abcam; 1:100), F4/80 (MCA497RT, Serotec, Oxford, UK; 1:100) and MHCII (ab157210, Abcam; 1:100), all diluted in TBS, BSA and Triton X-100. After a rinse in TBS (four times for 5 min), binding sites of primary antibodies were visualized by corresponding Alexa-Fluor-488-, Alexa-Fluor-568-, or Alexa-Fluor-647-tagged antisera (1:500; Invitrogen, Karlsruhe, Germany) in TBS, containing 1% BSA and 0.5% Triton X-100 (1 h at RT), followed by another rinse in TBS (four times for 5 min). The GFP signal of the transgenic animals was enhanced using a goat anti-GFP antibody (600-101-215S, Rockland, Limerick, PA, USA; 1:500). Some of the slides received additional nuclear staining using 4′,6-diamidino-2-phenylindol dihydrochloride (DAPI). For that, slides were incubated for 10 min (1:4000, stock 1 mg/ml, VWR, Vienna, Austria) followed by a rinse in PBS (three times for 5 min). All slides were embedded in Fluoromount™ Aqueous Mounting Medium (Sigma-Aldrich). Negative controls were performed by omission of the primary antibodies during incubation and resulted in absence of immunoreactivity.

### *In situ* phagocytosis assay

Rat flexor tendons (*n*=3) were freshly isolated and halved lengthwise by a scalpel. The tendons were placed in a 12-well cell culture dish with the cut surface pointing upwards in minimum essential medium supplemented with 10% FBS, exposing the tendon proper. pHrodo™ Green *S. aureus* Bioparticles™ Conjugate for Phagocytosis (P35367, Thermo Fisher Scientific, Massachusetts, MA, USA) were added to the tendons at a final concentration of 100 µg/ml. These particles are non-fluorescent outside the cell at neutral pH but fluorescent (488 nm) at acidic pH, such as in phagosomes, thus allowing the identification of cells with phagocytic activity.

After 24 h, the tendons were counterstained with DAPI for 5 min and analyzed by confocal microscopy.

### Confocal imaging

Confocal imaging was performed using an LSM1 700 confocal microscope (Zeiss) equipped with 405-nm (5 mW fiber output), 488-nm (10 mW fiber output), 555-nm (10 mW fiber output) and 639-nm (5 mW fiber output) diode lasers, a main dichroic beam splitter URGB and a gradient secondary beam splitter for LSM 700, using a 10× EC Plan-Neofluar (10×/0.3) or a 20× Plan-Apochromat (20×/0.8) objective (Zeiss, Munich, Germany). Image acquisition was performed with ZEN 2010 (Zeiss), and image dimensions were 1024×1024 pixels with an image depth of 16 bit. Two times averaging was applied during image acquisition. Laser power and gain were adjusted to avoid saturation of single pixels. All images were taken using identical microscope settings based on the secondary antibody control staining.

### qRT-PCR

Total RNA was isolated from tendon-like constructs (*n*=5 animals, two constructs each) using TRI^®^ Reagent (Sigma-Aldrich) according to the manufacturer's protocol. RNA yield was quantified using a Nanodrop 2000C (Thermo Fisher Scientific, Vienna, Austria) and RNA integrity was verified using an Experion Automated Electrophoresis system (Bio-Rad, Munich, Germany). A minimum requirement of the RNA quality indicator (RQI) >7.5 was chosen.

qRT-PCR was performed as described by Lehner et al. (2016) using TaqMan^®^ assays from Integrated DNA Technologies (Coralville, IA, USA) targeting all genes listed in Table 1. Amplification conditions were 50°C for 2 min, 95°C for 10 min, followed by 40 cycles of 95°C for 15 s and 60°C for 1 min. All samples were run in duplicate. CQ values were analyzed using qBasePlus v. 2.4 (Biogazelle NV, Zwijnaarde, Belgium) and normalized relative quantities were calculated by normalizing the data to the expression of previously validated endogenous control genes as described by Vandesompele et al. (2002). As housekeeping genes, eukaryotic translation initiation factor 2B subunit alpha (*Eif2b1*), polymerase (RNA) II (DNA directed) polypeptide A (*Polr2a*) and tyrosine 3-monooxygenase/tryptophan 5-monooxygenase activation protein zeta (*Ywhaz*) were used. The normalized quantities were then determined for the candidate genes scaled against the expression values determined for the controls to generate fold changes in expression.

**Table 1.**
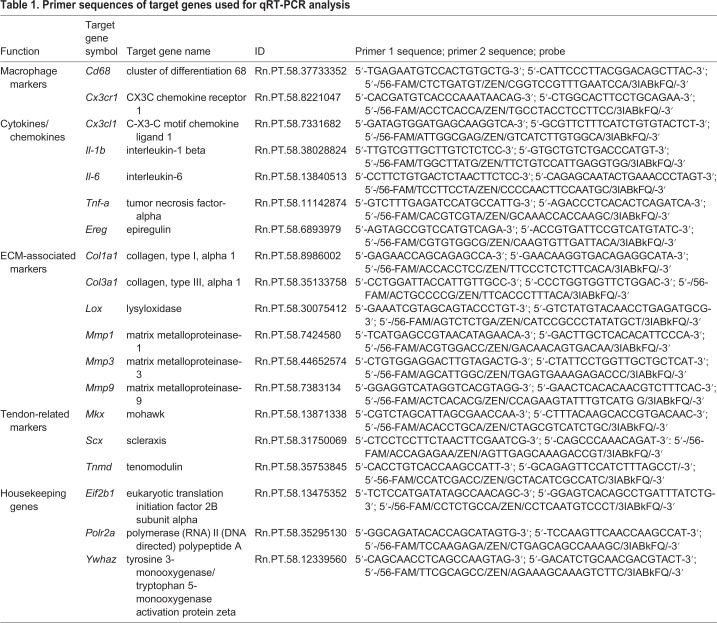
**Primer sequences of target genes used for qRT-PCR analysis**

### Western blot analysis

First, 10-15 μg of total protein of the tendon-like constructs' lysate was separated on 10-12% SDS-polyacrylamide gels in Laemmli buffer. Proteins were then transferred to a PVDF membrane (Bio-Rad) using 15.6 mM Tris base, 120 mM glycine and 20% methanol for 1.5 h at 90 V and 4°C. Membranes were blocked in 5% non-fat dry milk powder or 5% BSA hydrolysate in TBS with 0.5% Tween-20 overnight at 4°C. Immunodetection was performed using primary antibodies recognizing epiregulin and CX3CL1, and secondary horseradish peroxidase (HRP)-labeled goat anti-rabbit antibodies (Bio-Rad). Bands were visualized using the Clarity™ Western ECL substrate from Bio-Rad (170-5060). Band intensities of at least three individual experiments were measured densitometrically and normalized to whole protein using the Image Lab Software 5.1 from Bio-Rad.

### Migration assay

In order to examine a potential role of Fkn present in tendon cells on migratory processes, we performed a migration assay using AZD 8797 (Axon Medchem, Groningen, Netherlands), a selective, high-affinity small-molecule inhibitor of CX3CR1. To this end, we seeded rat TDSPCs on both uncoated and collagen-coated Petri dishes. Cells were grown to confluence and serum starved at 1% serum for 24 h in order to arrest proliferation. The cell monolayer was then scratched by a sterile 200-µm pipette tip and further cultivated in the presence and absence of the inhibitor. After 24 h, images were taken with a microscope and the distance between the wound margins was measured (Cory, 2011).

### Statistical analysis

All experiments were repeated at least three times. Statistical analyses were performed using GraphPad Prism v.5.04 (La Jolla, CA, USA). Numerical data are presented as means±s.d. One-way analysis of variance (ANOVA) for multiple comparisons and two-sample Student's *t*-test for pairwise comparisons were employed after confirming normal distribution of the data (D'Agostino and Pearson omnibus normality test). Non-parametric statistics were utilized when the above assumption was violated, and consequently Kruskal–Wallis test for multiple comparisons or Mann–Whitney test to determine two-tailed *P*-value samples was carried out. Statistical significance was set at α=0.05.

## Supplementary Material

Supplementary information
