# Supplementary material for: Tenophages: a novel macrophage-like tendon cell population expressing CX3CL1 and CX3CR1
Source: Dis Model Mech. 2019 Dec 16;12(12):dmm041384. doi: 10.1242/dmm.041384 (PMC6918766; doi:10.1242/dmm.041384)
Supplement: Supplementary information [file dmm-12-041384-s1.pdf]

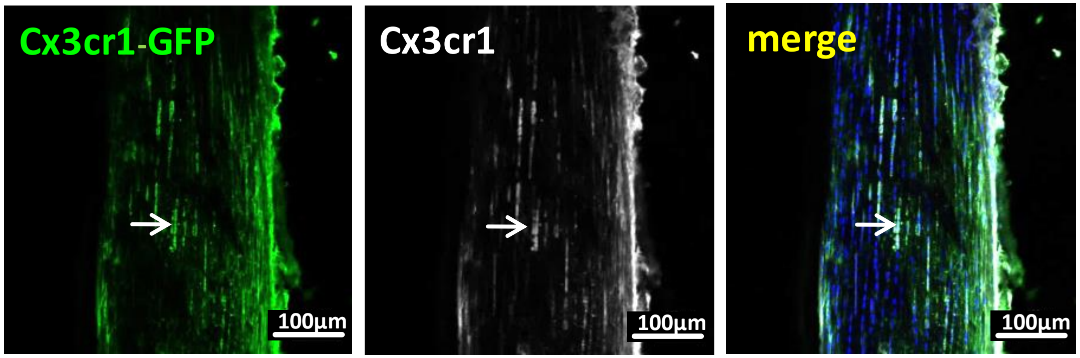

Fig. S1
Longitudinal cryo-sections of Achilles tendons from Cx3cr1-GFP transgenic mice co-stained with an antibody directed against the Cx3cr1 protein shows a high degree of overlap (merge), confirming the expression pattern of the Cx3cr1-GFP protein.

Disease Models & Mechanisms • Supplementary information

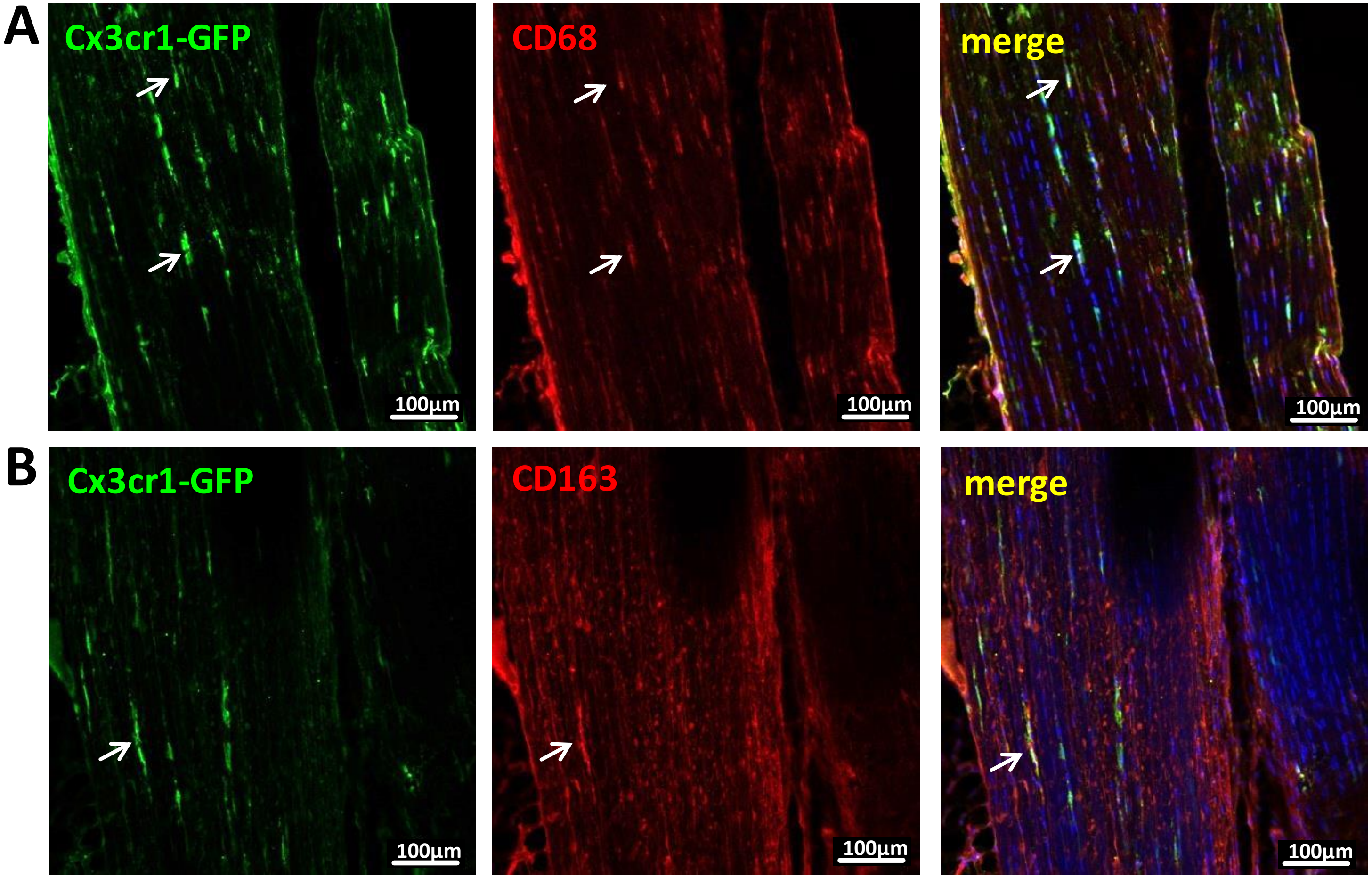

Fig. S2

Doublelabelling of longitundinal cryo-sections of Achilles tendons from Cx3cr1-GFP transgenic mice with antibodies directed against the macrophage-related markers CD68 and CD163 revealed co-expression of these markers with the Fkn receptor (see arrows).

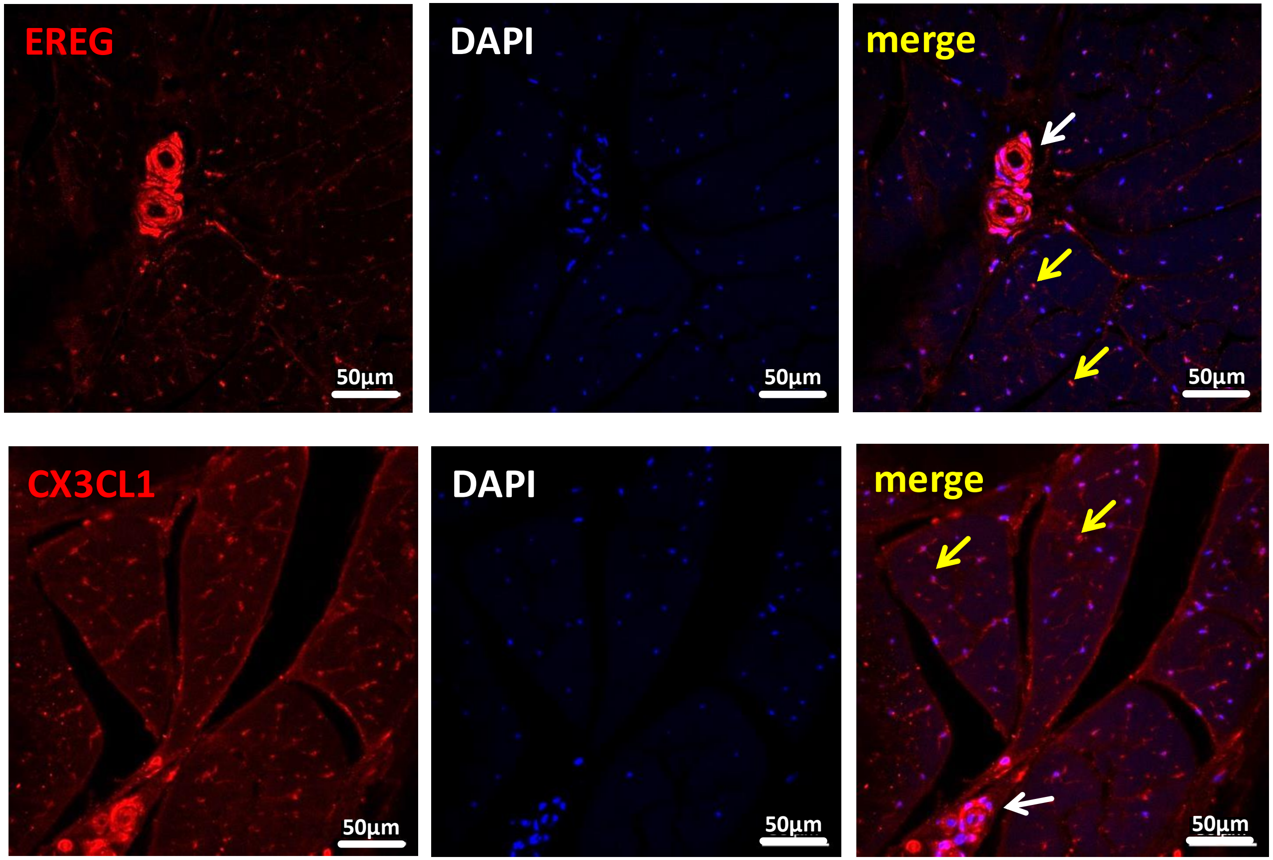

Fig. S3

Cross sections of an intact human semitendinosus tendon (♂, 22 years) demonstrating the expression of CX3CL1 and epiregulin in the perivascular region (see white arrows) and the tendon proper (yellow arrows).

Disease Models & Mechanisms • Supplementary information
